# Impact of body mass index on perioperative outcomes and short-term recovery profiles of single-incision laparoscopic appendectomy: a propensity score-matched analysis

**DOI:** 10.3389/fmed.2026.1903116

**Published:** 2026-09-15

**Authors:** Dake Liu, Yubin Zhang, Fei Xue, Kehua Shi, Yu Cheng, Jianbin Gu

**Affiliations:** Department of Hepatobiliary Surgery, Shijiazhuang People’s Hospital, Shijiazhuang, China

**Keywords:** appendectomy, appendicitis, body mass index, pain, recovery, single-port

## Abstract

**Objective:**

This study aimed to evaluate the association between stratified body mass index (BMI) and the intraoperative outcomes and short-term postoperative recovery profiles of patients undergoing single-incision laparoscopic appendectomy (SILA), utilizing a propensity score-matched (PSM) analysis to minimize baseline selection bias and confounding clinical factors.

**Methods:**

Clinical data of 282 patients who underwent SILA between January 2024 and January 2026 were retrospectively analyzed, comprising 93 patients in the normal weight group, 97 in the overweight group, and 92 in the obesity group. To control for baseline confounding, a 1:1 nearest-neighbor PSM with a strict caliper of 0.02 was performed based on preoperative covariates, including age, gender, comorbidities, history of abdominal surgery, preoperative delay, and pathological diagnosis. Intraoperative parameters and postoperative recovery profiles were subsequently compared among the matched groups.

**Results:**

After 1:1 propensity score matching, a well-balanced cohort was successfully analyzed, with all preoperative baseline variables closely aligned (all *P* > 0.05). In the matched cohort, appendiceal length and width showed no statistical differences among the stratified groups (*P* > 0.05). Importantly, no patients required conversion to open surgery. However, higher BMI was associated with a stepwise prolongation of operative time (*P* = 0.002) and greater intraoperative blood loss (*P* < 0.001). Postoperatively, the time to first flatus was longer in patients with higher BMI (*P* < 0.001). Furthermore, higher BMI was associated with elevated postoperative pain scores (*P* < 0.001) and a higher incidence of gastrointestinal adverse reactions, including nausea/vomiting (*P* = 0.008), abdominal distension (*P* = 0.001), and diarrhea (*P* = 0.006).

**Conclusion:**

While single-incision laparoscopic appendectomy remains safe and technically feasible, an elevated BMI was associated with impaired operative efficiency, more intense early postoperative pain, and slower short-term gastrointestinal recovery. Clinicians should fully weigh these hidden recovery costs and metabolic stress profiles during perioperative management for overweight and obese patients.

## Highlights

- 1:1 propensity score matching effectively balanced baseline demographic and clinical confounders across stratified BMI groups.
- Elevated BMI was associated with prolonged operative duration, increased intraoperative blood loss, and delayed first flatus.
- Acute postoperative pain scores and rescue analgesic use were higher in groups with higher BMI, showing a stepwise pattern.
- Postoperative hospital stays, laboratory inflammatory markers, and cumulative inpatient costs remain statistically uniform.
- SILA may benefit from cautious patient selection and optimized perioperative pathways in obese populations.

## 1 Introduction

Acute appendicitis represents one of the most prevalent surgical emergencies worldwide, necessitating prompt intervention to prevent progression to perforation, abscess formation, or diffuse peritonitis (1, 2). Over the past few decades, conventional multi-port laparoscopic appendectomy (LA) has firmly established itself as the gold standard of care due to its well-documented clinical advantages, including reduced postoperative pain, lower surgical site infection rates, and accelerated recovery compared to open laparotomy (3, 4). In the continuous pursuit of minimizing surgical trauma and maximizing cosmetic outcomes, single-incision laparoscopic appendectomy (SILA) has emerged as a compelling evolutionary alternative (5). By consolidating all working instruments and the camera port into a single transumbilical incision, SILA offers superior cosmetic satisfaction with virtually invisible scarring, while theoretically minimizing abdominal wall trauma, parietal pain, and incision-related complications (6, 7).

Concurrently, the global burden of overweight and obesity has escalated dramatically, presenting unique anatomical and physiological challenges to surgical teams (8, 9). While conventional multi-port laparoscopic approaches are widely preferred for patients with an elevated body mass index (BMI) due to superior deep-field exposure compared to open surgery, the clinical feasibility and real-world efficiency of SILA in this specific population remain a subject of intense debate (10). The inherent technical limitations of SILA—namely, severe instrument crowding, cross-clashing, and the loss of standard triangulation—are significantly magnified by a thick abdominal wall and abundant intra-abdominal or omental fat (11). These hyperplastic adipose tissues heavily compromise optical visualization, impede effective organ retraction, and increase the physical workload of the operator, potentially translating into prolonged operative durations, increased minor hemorrhage, or a higher risk of conversion to multi-port or open surgery (12, 13).

Despite these theoretical and practical concerns, current literature evaluating the safety, tolerance, and recovery kinetics of SILA in overweight and obese populations has reported varied findings, and a definitive clinical consensus has yet to be established (14). Furthermore, many historical studies are fundamentally restricted by small sample sizes, a lack of fine-grained stratification across distinct BMI sub-cohorts, or a failure to control for crucial confounding baselines—such as patient age, comorbidities, and baseline appendiceal inflammatory severity—thereby introducing profound retrospective selection bias. To address these methodologic limitations, this retrospective cohort study utilized two sequential 1:1 propensity score-matched (PSM) procedures with a caliper width of 0.02. By comprehensively balancing preoperative baseline covariates, we aimed to evaluate the association between stratified BMI and the intraoperative outcomes and short-term gastrointestinal recovery profiles of patients undergoing SILA within a high-volume minimally invasive center.

## 2 Methods

### 2.1 Study design and setting

This study was designed as a single-center, retrospective cohort study to evaluate the impact of BMI on the perioperative efficiency and short-term clinical outcomes of patients undergoing SILA. The study was conducted at Shijiazhuang People's Hospital. Clinical and electronic medical records of patients who were diagnosed with appendicitis (including acute and chronic types) and successfully managed with SILA between January 2024 and January 2026 were comprehensively reviewed. The final allocation of these eligible patients into the three comparative groups is schematically presented in the patient flowchart shown in Figure 1.

**Figure 1:**
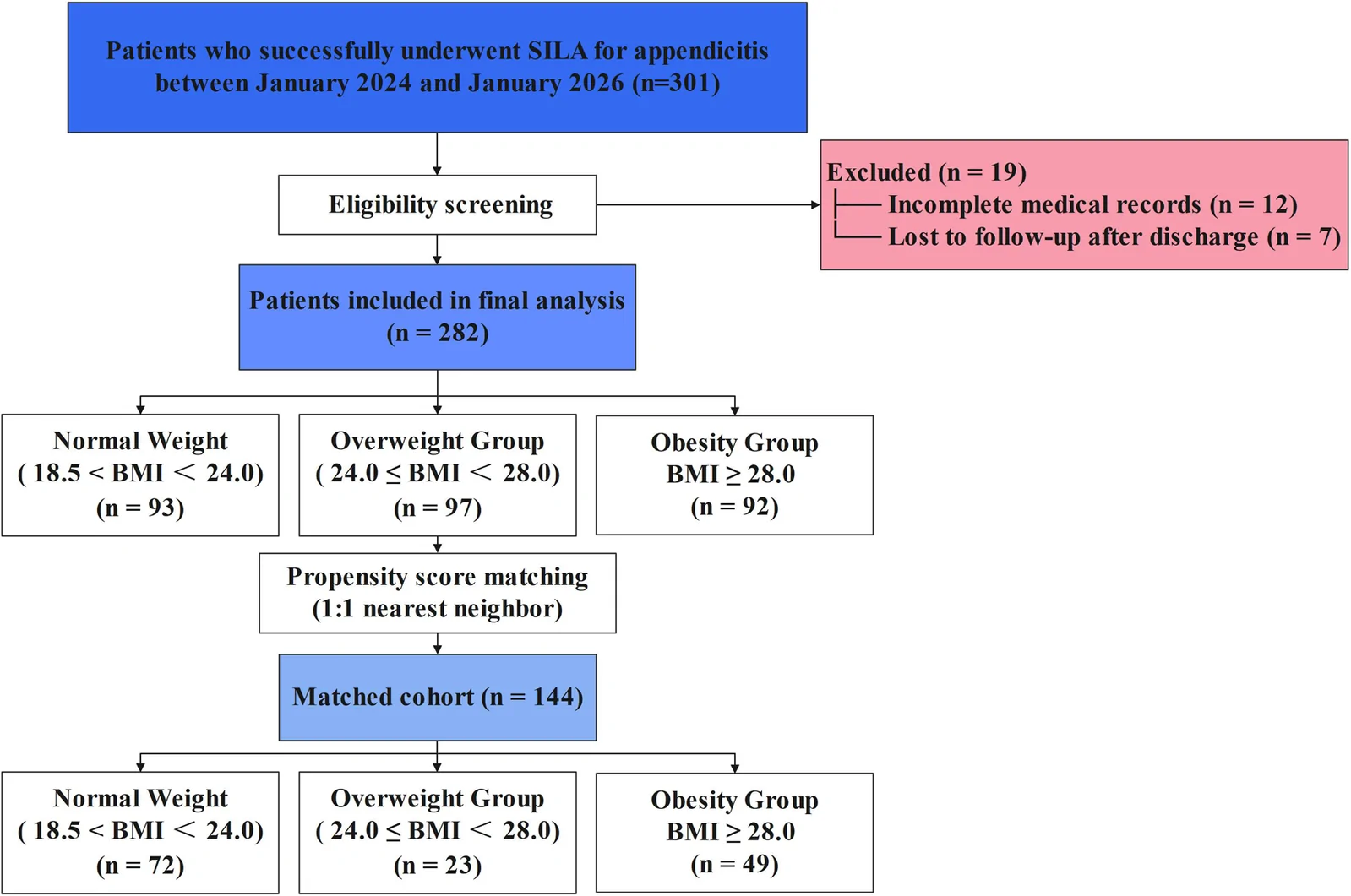
Flowchart of the study.

All surgical procedures were performed by a highly skilled and experienced medical team. The primary surgeons had each completed over 200 SILA procedures prior to the study period, and the center performs approximately 150 SILA cases annually, ensuring technical standardization and minimizing operator-dependent bias. This study was formally reviewed and ethically approved by the Medical Research Ethics Committee of Shijiazhuang People's Hospital (Approval No: 2025-027). The entire study protocol was carried out in strict accordance with the relevant guidelines, regulatory standards, and the ethical tenets of the Declaration of Helsinki. During the preoperative consultation, comprehensive clinical information and potential surgical outcomes were thoroughly provided to all patients and their families, who subsequently and voluntarily signed written informed consent documents pertaining to the surgical treatment. Furthermore, this study has been reported in line with the STROCSS guidelines (15).

According to the Guidelines for the Prevention and Control of Overweight and Obesity in Chinese Adults, patients enrolled in this cohort were precisely stratified into three sub-groups based on their calculated preoperative BMI profiles:

- Normal Weight Group: Patients with a BMI ranging from 18.5 kg/m^2^ to <24.0 kg/m^2^.
- Overweight Group: Patients with a BMI ranging from 24.0 kg/m^2^ to ≤28.0 kg/m^2^.
- Obesity Group: Patients with a BMI exceeding or equal to 28.0 kg/m^2^.

### 2.2 Inclusion and exclusion criteria

#### 2.2.1 Inclusion criteria

(1) Diagnosis of appendicitis was confirmed according to established criteria (16);
(2) Complete clinical data were available for analysis;
(3) The patient is able to cooperate with clinical treatment;
(4) No other abdominal surgeries were performed during hospitalization.

#### 2.2.2 Exclusion criteria

(1) Patients with a BMI <18.5 kg/m^2^ (17);
(2) Patients with severe coagulation disorders or poor cardiopulmonary function who are unable to tolerate surgery or anesthesia;
(3) Patients unable to comply with clinical treatment;
(4) Patients unable to adhere to postoperative follow-up;
(5) Conditions of the female reproductive system, including pregnancy.

### 2.3 Surgical equipment and platforms

All single-incision laparoscopic procedures in this study were executed utilizing two standardized visualization platforms: the high-definition Karl Storz laparoscopic system (Karl Storz Endoscopy-America Inc., Tuttlingen, Germany) and the DPM electronic laparoscopic system (Beijing Precision Digital Medical Technology Co., Ltd., Beijing, China).

To facilitate the transumbilical single-port access, a disposable multi-channel single-incision laparoscopic surgical device (Production Number: 104279; Shenzhen Mindray Bio-Medical Electronics Co., Ltd., Shenzhen, China) was deployed in all cases, ensuring standard working channel isolation and pneumatic stability during the operation.

### 2.4 Surgical procedure

All SILA procedures were completed under general anesthesia with endotracheal intubation, with the patient placed in the supine position. Following strict aseptic preparation, a 20–25 mm transumbilical incision was carefully made to gain sequential access to the peritoneal cavity. The disposable multi-channel single-port device was then inserted, and a CO_2_ pneumoperitoneum was established at a standardized pressure of 11–13 mmHg. Monopolar electrocautery was primarily utilized for dissection throughout the procedure. After clear identification of the appendiceal base, the mesoappendix was windows-opened and skeletonized through a hypervascular-free area on the mesenteric side, followed by secure ligation with Hem-o-lok clips and subsequent transection. Double ligation was meticulously executed 0.5 cm from the appendiceal base using a combination of an endoloop snare and Hem-o-lok clips. The resected specimen was encapsulated and retrieved through the transumbilical port channel. Intraperitoneal warm saline irrigation was selectively administered based on the presence of inflammatory purulent exudate. Absolute hemostasis was rigorously confirmed prior to withdrawing the laparoscopic instruments, after which the CO_2_ gas was fully evacuated. Finally, anatomical layered closure of the abdominal fascial defect and subcuticular tissues was performed using absorbable sutures to optimize wound healing and minimize incisional hernia risks.

### 2.5 Postoperative management

An evidence-based enhanced recovery after surgery (ERAS) pathway was systematically implemented, incorporating standardized multimodal postoperative care for all patients (18, 19). Continuous monitoring of vital signs was maintained until stable clinical baselines were achieved. Postoperatively, meticulous administration of fluid, electrolyte, and nutritional support was performed alongside tailored pain management as required.

Pain intensity was assessed using the Numerical Rating Scale (NRS, 0–10). To ensure comprehensive capture of the pain experience, NRS scores were systematically recorded by trained nursing staff at 1, 3, 7, 9, 12, and 24 h postoperatively. For statistical analysis, the peak NRS score—the highest value recorded across all six time points—was used as the definitive measure of pain intensity for each patient. Pain scores were documented by ward nurses who were not involved in the study design or data analysis. Complete blinding to BMI group was not feasible due to the nature of the study. In line with the expected mild pain profile after single-incision laparoscopic appendectomy, all patients received on-demand analgesia only. Ketorolac tromethamine injection (1 mL: 30 mg) was administered upon patient request.

On the first postoperative day, routine hematological and biochemical screenings—including complete blood count, liver competence, and renal function panels—were performed to monitor the acute recovery trajectory. Patients were encouraged to ambulate twice daily, oral intake was advanced in a stepwise manner after first flatus, and discharge was determined by standardized clinical criteria including adequate oral intake, satisfactory pain control, independent ambulation, absence of fever, and passage of flatus. Postoperative gastrointestinal outcomes—including time to first flatus, nausea, vomiting, abdominal distension, and diarrhea—were extracted from routine nursing records and twice-daily surgical team assessments documented in the electronic medical record. Systemic inflammatory and stress states were monitored by tracking febrile symptoms, with peak body temperatures recorded during the early postoperative phase.

One month post-discharge, telephone follow-up was conducted by two designated staff members to assess wound healing, pain, and any need for readmission; 7 patients were lost to this follow-up.

### 2.6 Observation outcomes and measurement methods

Clinical parameters collected in this study were categorized into preoperative, intraoperative, and postoperative variables:

- Preoperative Variables: Baseline demographics and clinical characteristics included age, gender, previous history of abdominal surgery, and comorbidities (hypertension and diabetes). Preoperative clinical delay was classified into 0–24 h, 24–72 h, and >72 h. Preoperative laboratory parameters comprised alanine aminotransferase (ALT), albumin (Alb), serum creatinine (Cr), C-reactive protein (CRP), total white blood cell count (WBC), and neutrophil count (NEUT). Pathological diagnosis was stratified into acute simple appendicitis, acute suppurative appendicitis, acute gangrenous appendicitis, and chronic appendicitis.
- Intraoperative Variables: Intraoperative outcomes evaluated were operative time (from initial skin incision to final dermal closure), estimated intraoperative blood loss, and appendiceal gross dimensions (length and width). The incidence of technical conversion to multi-port laparoscopy or open surgery was monitored.
- Postoperative Variables: Clinical recovery metrics consisted of the time to first flatus (exhaust time) and specific adverse reactions, including nausea/vomiting, coughing/expectoration, abdominal distension, and diarrhea. Systemic fever occurrence was recorded and stratified into low-grade fever (37.4 °C–38.0 °C), moderate fever (38.1 °C–39.0 °C), and high fever (39.1 °C–41.0 °C). Acute post-surgical pain profiles were evaluated via the postoperative 0–24 h pain score and the objective rate of rescue analgesic consumption within 24 h. Additionally, laboratory markers (WBC and NEUT counts) on postoperative day 1, postoperative hospital stay, and cumulative inpatient costs were recorded.

### 2.7 Statistical analysis

All statistical computations in this study were executed utilizing SPSS 25.0 software, with statistical significance for all hypothesis tests defined as a two-tailed *P* < 0.05. Continuous variables were initially evaluated for normality utilizing the Shapiro–Wilk test, with normally distributed data expressed as mean ± standard deviation $\left(\bar{x}\pm s\right)$ and non-normally distributed data (comprising intraoperative blood loss, appendiceal dimensions, and postoperative hospital stay) presented as median and interquartile range (M[P25, P75]). Categorical and ordinal variables were presented as frequencies and percentages (n [%]). For continuous data across the three stratified BMI groups, one-way Analysis of Variance (ANOVA) was executed for normally distributed variables, followed by the Least Significant Difference (LSD) or Bonferroni test for *post-hoc* pairwise comparisons, while non-normally distributed continuous data and ordinal variables (including preoperative total delay and postoperative fever stratification) were analyzed utilizing the non-parametric Kruskal–Wallis H test. Categorical data were evaluated using the Pearson Chi-square test or Fisher's exact test where appropriate.

To control for baseline selection bias and balance confounding variance, two sequential 1:1 nearest-neighbor PSM procedures were performed without replacement, using the normal-weight group as the common reference. In the first step, normal-weight patients were matched 1:1 with obese patients. In the second step, the remaining unmatched normal-weight patients were matched 1:1 with overweight patients. All preoperative baseline variables—including demographics (age and gender), history of abdominal surgery, metabolic comorbidities (hypertension and diabetes), preoperative total delay, laboratory parameters (ALT, Alb, Cr, CRP, WBC, and NEUT), and pathological diagnosis—were fully incorporated into the logistic regression model to calculate propensity scores for each matching step. The caliper width was strictly set at 0.02 for both procedures. Covariate balance was assessed after each matching step using standardized mean differences (SMDs). SMD was calculated as the absolute difference in means or proportions divided by the pooled standard deviation, with an SMD < 0.10 considered to indicate adequate balance.

## 3 Results

### 3.1 Patient baseline characteristics

Table 1 details the preoperative baseline characteristics and demographic distributions of the initially enrolled cohort before PSM. A total of 282 patients who underwent SILA for appendicitis were evaluated, comprising 93 patients in the Normal Weight Group, 97 patients in the Overweight Group, and 92 patients in the Obesity Group. Prior to matching, statistically significant baseline differences were observed among the three groups in terms of age (*P* = 0.020) and preoperative albumin levels (*P* = 0.030). Specifically, patients in the overweight category (36.47 ± 17.53 years) and the obese category (37.98 ± 16.52 years) were significantly older than those in the normal weight category (31.21 ± 12.37 years). Additionally, preoperative albumin was significantly lower in the obesity group (37.32 ± 1.49 g/L) compared to the normal weight group (37.86 ± 1.94 g/L) and the overweight group (38.01 ± 2.06 g/L). Other preoperative covariates—including gender, history of abdominal surgery, hypertension, diabetes, preoperative total delay, liver and renal functions (ALT and Cr), systemic inflammatory markers (CRP, WBC, and NEUT), and pathological diagnosis distribution—demonstrated no statistical differences before matching (all *P* > 0.05) as displayed in Table 1.

**Table 1:** Preoperative baseline characteristics of patients across body mass Index categories before propensity score matching.

Characteristics	Normal Weight Group (*n* = 93)	Overweight group (*n* = 97)	Obesity Group (*n* = 92)	Statistical test values	*P* value
Gender [n(%)]				3.939	0.140
Men	39 (41.94%)	48 (49.48%)	52 (56.21%)		
Women	54 (58.06%)	49 (50.52%)	40 (43.48%)		
Age ($\bar{x}\pm s$, years)	31.21 ± 12.37^a^	36.47 ± 17.53^b^	37.98 ± 16.52^b^	3.979	0.020
Previous history of abdominal surgery [n(%)]				0.231	0.891
Yes	12 (12.90%)	13 (13.40%)	14 (15.22%)		
No	81 (87.10%)	84 (86.60%)	78 (84.78%)		
Hypertension [n(%)]				0.907	0.635
Yes	7 (7.53%)	11 (11.34%)	10 (10.87%)		
No	86 (92.47%)	86 (88.66%)	82 (89.13%)		
Diabetes [n(%)]				2.213	0.201
Yes	4 (4.30%)	6 (6.19%)	10 (10.87%)		
No	89 (95.70%)	91 (93.81%)	82 (89.13%)		
Preoperative total delay [n(%)]				3.265	0.515
0–24h	25 (26.88%)	37 (38.14%)	29 (31.52%)		
24–72h	20 (21.51%)	20 (20.62%)	18 (19.57%)		
>72h	48 (51.56%)	40 (41.24%)	35 (48.91%)		
ALT $\left(\bar{x}\pm s,\;U/L\right)$	34.55 ± 10.52	36.39 ± 10.90	37.75 ± 10.63	2.091	0.125
Alb $\left(\bar{x}\pm s,\;g/L\right)$	37.86 ± 1.94^a^	38.01 ± 2.06^a^	37.32 ± 1.49^b^	3.561	0.030
Cr $\left(\bar{x}\pm s,\;\mu \mathrm{mol}/L\right)$	54.78 ± 10.74	52.77 ± 7.65	51.67 ± 8.02	0.478	0.620
CRP $\left(\bar{x}\pm s,\;\mathrm{mg}/L\right)$	46.63 ± 16.55	50.85 ± 17.60	51.65 ± 16.39	2.376	0.095
WBC $\left(\bar{x}\pm s,\;10^{9}/L\right)$	9.17 ± 1.88	9.10 ± 1.02	9.28 ± 1.82	0.268	0.765
NEUT $\left(\bar{x}\pm s,\;10^{9}/L\right)$	8.02 ± 1.92	7.91 ± 2.02	8.45 ± 1.38	2.360	0.096
Pathological diagnosis [n(%)]				7.933	0.243
Acute simple appendicitis	44 (47.31%)	35 (36.08%)	39 (42.39%)		
Acute suppurative appendicitis	21 (22.58%)	26 (26.80%)	18 (19.57%)		
Acute gangrenous appendicitis	8 (8.60%)	17 (17.53%)	9 (9.78%)		
Chronic appendicitis	20 (21.51%)	19 (19.59%)	26 (28.26%)		

Please note the superscript letters (a, b) for each number; if the same letter appears, it indicates no statistical significance between them.

Following the two-step 1:1 nearest-neighbor PSM procedure described in the Methods, we obtained a well-balanced matched cohort of 144 patients (72 normal-weight, 23 overweight, and 49 obese), with all post-matching SMDs below 0.10 confirming adequate covariate balance, as documented in Table 2. The reduction in SMDs after matching is shown in Figure 2. This matched sample consisted of 72 patients in the Normal Weight Group and 72 patients in the elevated BMI pool, which included 23 patients in the Overweight Group and 49 patients in the Obesity Group. Following the application of the 0.02 caliper, all baseline covariates were adequately balanced across groups, as confirmed by post-matching SMDs below 0.10, shown in Table 2. Post-matching analyses revealed that differences in age (*P* = 0.287) and preoperative albumin (*P* = 0.099) were successfully neutralized. All other demographic parameters, comorbidities, preoperative delay intervals, acute inflammatory markers, and specific pathological types achieved complete statistical equilibrium (all *P* > 0.05) as indicated in Table 2, establishing an optimal, unbiased foundation for subsequent comparative assessments of clinical outcomes.

**Table 2:** Preoperative baseline characteristics of patients across body mass Index categories after propensity score matching.

Characteristics	Normal Weight Group (*n* = 72)	Overweight Group (*n* = 23)	Obesity Group (*n* = 49)	Statistical test values	*P* value	SMD (Before matching)	SMD (After matching)
Gender [n(%)]				1.885	0.390	0.152/0.288	0.014/0.031
Men	25 (34.72%)	8 (34.78%)	22 (44.90%)				
Women	47 (65.28%)	15 (65.22%)	27 (55.10%)				
Age ($\bar{x}\pm s$, years)	32.61 ± 12.97	28.57 ± 16.58	34.53 ± 16.53	1.258	0.287	0.347/0.464	0.042/0.055
Previous history of abdominal surgery [n(%)]				0.204	1.000	0.015/0.067	0.022/0.011
Yes	9 (12.50%)	2 (8.70%)	6 (12.24%)				
No	63 (87.50%)	21 (91.30%)	43 (87.76%)				
Hypertension [n(%)]				1.161	0.533	0.131/0.116	0.038/0.045
Yes	7 (9.72%)	3 (13.04%)	3 (6.12%)				
No	65 (90.28%)	20 (86.96%)	46 (93.88%)				
Diabetes [n(%)]				1.093	0.544	0.085/0.250	0.019/0.033
Yes	2 (2.78%)	1 (4.35%)	3 (6.12%)				
No	70 (97.22%)	22 (95.65%)	46 (93.88%)				
Preoperative total delay [n(%)]				2.196	0.700	0.208/0.053	0.051/0.042
0–24h	21 (29.17%)	7 (30.43%)	13 (26.53%)				
24–72h	18 (25.00%)	3 (13.04%)	14 (28.57%)				
＞72h	33 (45.83%)	13 (56.52%)	22 (44.90%)				
ALT $\left(\bar{x}\pm s,\;U/L\right)$	34.97 ± 10.75	33.17 ± 8.81	35.98 ± 11.27	0.545	0.581	0.172/0.302	0.048/0.061
Alb $\left(\bar{x}\pm s,\;g/L\right)$	37.69 ± 1.95	38.41 ± 2.04	37.40 ± 1.57	2.351	0.099	0.075/0.312	0.035/0.052
Cr $\left(\bar{x}\pm s,\;\mu \mathrm{mol}/L\right)$	53.15 ± 10.51	51.30 ± 8.37	51.67 ± 8.02	0.386	0.680	0.216/0.328	0.063/0.047
CRP $\left(\bar{x}\pm s,\;\mathrm{mg}/L\right)$	50.57 ± 15.54	45.61 ± 16.71	50.81 ± 15.18	1.020	0.363	0.247/0.305	0.057/0.064
WBC $\left(\bar{x}\pm s,\;10^{9}/L\right)$	9.05 ± 1.94	9.09 ± 1.22	8.83 ± 1.66	0.280	0.756	0.046/0.059	0.012/0.024
NEUT $\left(\bar{x}\pm s,\;10^{9}/L\right)$	7.86 ± 1.88	7.64 ± 1.92	8.34 ± 1.42	1.649	0.196	0.056/0.257	0.015/0.038
Pathological diagnosis [n(%)]				3.548	0.738	0.229/0.099	0.061/0.075
Acute simple appendicitis	33 (45.83%)	12 (52.17%)	30 (61.22%)				
Acute suppurative appendicitis	13 (18.06%)	5 (21.74%)	8 (16.33%)				
Acute gangrenous appendicitis	9 (12.50%)	2 (8.70%)	4 (8.16%)				
Chronic appendicitis	17 (23.61%)	4 (17.39%)	7 (14.29%)				

SMD, standardized mean difference. Values are shown as (Normal vs. Overweight)/(Normal vs. Obesity). “Before matching” SMDs were calculated from the original cohorts and “After matching” from the matched sub-cohorts (23 vs. 23; 49 vs. 49). SMD <0.10 indicates adequate covariate balance.

**Figure 2:**
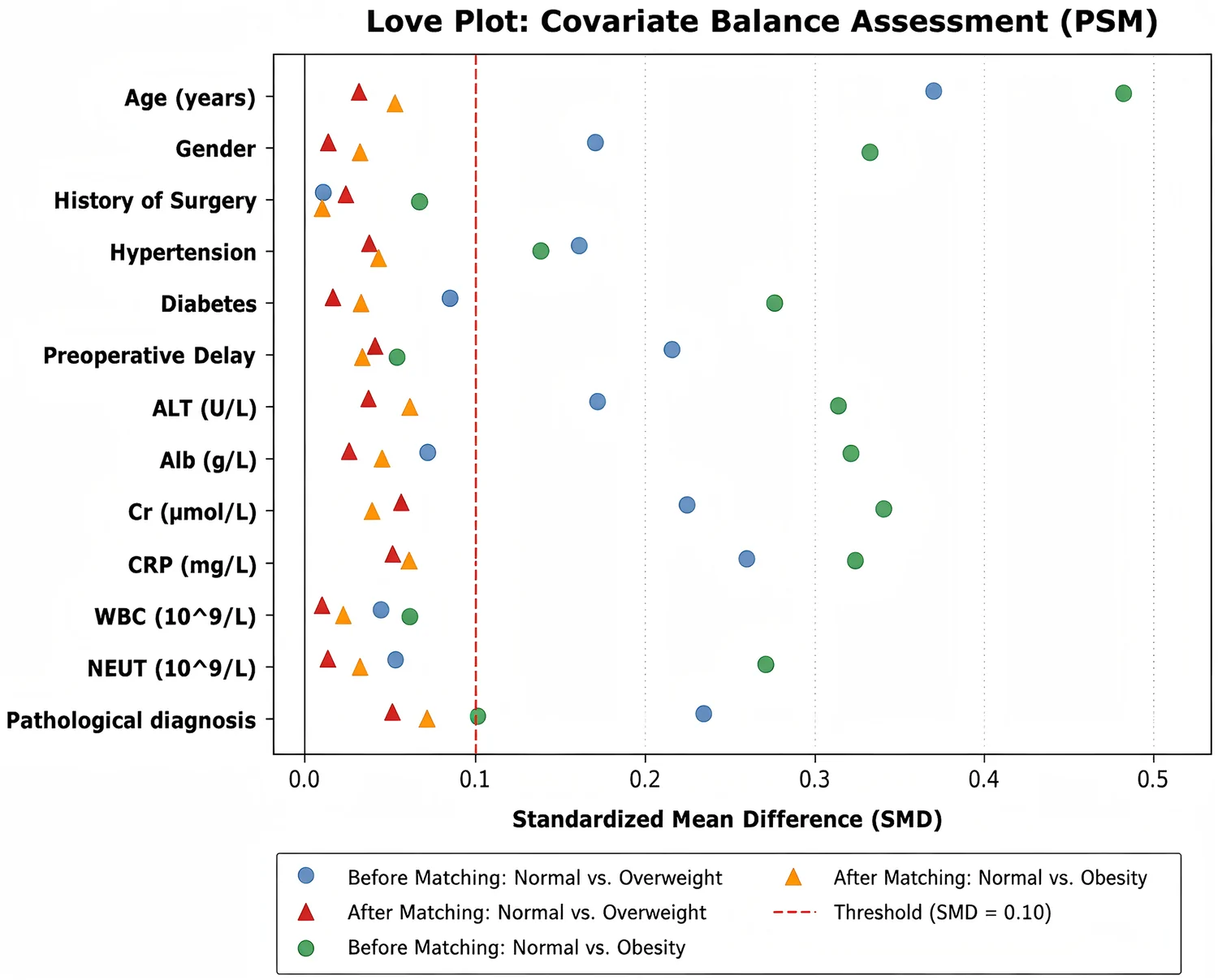
Standardized mean differences before and after propensity score matching for All baseline covariates.

### 3.2 Intraoperative outcomes

Table 3 delineates the comparison of intraoperative parameters across the three BMI categories before PSM. Prior to matching, statistically significant differences were observed among the groups regarding operative time (*P* = 0.000) and estimated intraoperative blood loss (*P* = 0.000). Specifically, the operative time was significantly shorter in the Normal Weight Group (57.42 ± 16.01 min) compared to the Overweight Group (65.63 ± 18.35 min) and the Obesity Group (67.35 ± 19.36 min). *Post-hoc* pairwise comparisons indicated that while the normal-weight cohort required significantly less operative time, no statistical difference was established between the overweight and obese cohorts. For intraoperative blood loss, the Obesity Group presented a significantly higher median value (5.00 [5.00, 10.00] mL) than both the Normal Weight Group (5.00 [2.00, 5.00] mL) and the Overweight Group (5.00 [5.00, 5.00] mL). Conversely, baseline appendiceal length (*P* = 0.217) and appendiceal width (*P* = 0.206) demonstrated no statistical differences across the three unadjusted groups.

**Table 3:** Comparison of intraoperative outcomes across body mass Index categories before propensity score matching.

Intraoperative outcomes	Normal Weight Group (*n* = 93)	Overweight group (*n* = 97)	Obesity Group (*n* = 92)	Statistical test values	*P* value
Blood loss [M(P25, P75), mL]	5.00 (2.00, 5.00)^a^	5.00 (5.00, 5.00)^a^	5.00 (5.00, 10.00)^b^	26.133	*P* < 0.001
Appendiceal length [M(P25, P75), cm]	7.00 (6.00, 8.00)	8.00 (6.00, 9.00)	8.00 (6.25, 9.00)	3.053	0.217
Appendiceal width [M(P25, P75), cm]	0.80 (0.60, 0.80)	0.80 (0.65, 0.80)	0.80 (0.60, 0.90)	3.162	0.206
Operative time $\left(\bar{x}\pm s,\;\min\right)$	57.42 ± 16.01^a^	65.63 ± 18.35^b^	67.35 ± 19.36^b^	8.121	*P* < 0.001

Please note the superscript letters (a, b) for each number; if the same letter appears, it indicates no statistical significance between them.

Following the execution of the PSM algorithm, the intraoperative outcomes for the well-balanced cohorts were evaluated and are detailed in Table 4. Post-matching analyses revealed that an elevated BMI remained significantly associated with a prolongation of operative time (*P* = 0.002). The operative time expanded stepwise from the Normal Weight Group (57.40 ± 16.82 min) to the Overweight Group (64.83 ± 17.65 min) and the Obesity Group (68.59 ± 17.33 min). *Post-hoc* analyses confirmed that the normal-weight controls maintained a significantly lower operative duration than both elevated BMI sub-cohorts. Similarly, intraoperative blood loss differences remained highly significant (*P* = 0.000), with the Obesity Group exhibiting increased blood loss (5.00 [5.00, 10.00] mL) compared to the normal-weight (5.00 [2.00, 5.00] mL) and overweight (5.00 [2.00, 5.00] mL) patient segments. Importantly, appendiceal length (*P* = 0.457) and appendiceal width (*P* = 0.123) showed complete statistical homogeneity across the matched cohorts, eliminating anatomical confounding variations. Furthermore, a zero conversion rate was achieved, as no patients required middle-course conversion to conventional multi-port laparoscopic or open surgery.

**Table 4:** Comparison of intraoperative outcomes across body mass Index categories after propensity score matching.

Intraoperative outcomes	Normal Weight Group (*n* = 72)	Overweight group (*n* = 23)	Obesity Group (*n* = 49)	Statistical test values	*P* value
Blood loss [M(P25, P75), mL]	5.00 (2.00, 5.00)^a^	5.00 (2.00, 5.00)^a^	5.00 (5.00, 10.00)^b^	18.530	*P* < 0.001
Appendiceal length [M(P25, P75), cm]	7.00 (6.00, 8.00)	7.00 (6.00, 9.00)	7.00 (6.00, 8.00)	1.567	0.457
Appendiceal width [M(P25, P75), cm]	0.80 (0.60, 0.80)	0.80 (0.70, 1.00)	0.80 (0.60, 0.90)	4.189	0.123
Operative time $\left(\bar{x}\pm s,\;\min\right)$	57.40 ± 16.82^a^	64.83 ± 17.65^b^	68.59 ± 17.33^b^	6.500	0.002

Please note the superscript letters (a, b) for each number; if the same letter appears, it indicates no statistical significance between them.

### 3.3 Postoperative recovery and complications

Table 5 details the comparison of postoperative recovery pathways, pain profiles, and short-term complication rates across the three BMI categories before PSM. Prior to matching, the time to first flatus demonstrated highly significant differences among the groups (*P* = 0.000), with the Normal Weight Group (16.92 ± 2.31 h) achieving faster gastrointestinal recovery than both the Overweight Group (19.15 ± 2.74 h) and the Obesity Group (19.67 ± 2.53 h). Postoperative gastrointestinal adverse reactions showed prominent variation, including nausea and vomiting (*P* = 0.024), abdominal distension (*P* = 0.002), and diarrhea (*P* = 0.020), which presented significantly higher incidence rates within the elevated BMI sub-cohorts. Conversely, the occurrence of coughing and expectoration did not differ statistically across the groups (*P* = 0.341). Systemic pyrexial stress was significantly more pronounced in patients with higher BMI values (*P* = 0.004), characterized by an increased clustering of low-grade and high-grade fevers in the obesity segment. The utilization rate of rescue painkillers within 24 h was significantly lower in the normal-weight controls (19.35%) compared to the overweight (34.02%) and obese (34.78%) patients (*P* = 0.033). Correspondingly, the acute postoperative 0–24 h pain score increased progressively from the Normal Weight Group (2.32 ± 1.29) to the Overweight Group (3.07 ± 1.47) and the Obesity Group (3.72 ± 1.49), revealing highly significant intergroup variance (*P* = 0.000). On the other hand, postoperative day 1 inflammatory markers—including white blood cell count (*P* = 0.288) and neutrophil count (*P* = 0.120)—as well as postoperative hospital stay (*P* = 0.134) and cumulative inpatient costs (*P* = 0.205) established complete statistical homogeneity among the unadjusted cohorts in Table 5.

**Table 5:** Comparison of postoperative recovery and complications across body mass Index categories before propensity score matching.

Postoperative outcomes	Normal Weight Group (*n* = 93)	Overweight group (*n* = 97)	Obesity Group (*n* = 92)	Statistical test values	*P* value
First exhaust time after surgery $\left(\bar{x}\pm s,\;h\right)$	16.92 ± 2.31^a^	19.15 ± 2.74^b^	19.67 ± 2.53^b^	30.840	*P* < 0.001
Postoperative adverse reactions [n(%)]					
Nausea and vomiting	6 (6.45%)^a^	17 (17.53%)^b^	18 (19.57%)^b^	7.462	0.024
Coughing and expectoration	1 (1.08%)	2 (2.06%)	4 (4.38%)	2.154	0.341
Abdominal distension	4 (4.30%)^a^	16 (16.49%)^b^	20 (21.74%)^b^	12.202	0.002
Diarrhea	1 (1.08%)^a^	6 (6.19%)^b^	10 (10.87%)^b^	7.838	0.020
Postoperative fever occurrence [n(%)]				18.178	0.004
No fever	77 (82.80%)	72 (74.23%)	61 (66.30%)		
Low-grade fever	8 (8.60%)	11 (11.34%)	21 (22.83%)		
Moderate fever	7 (7.53%)	13 (13.40%)	4 (4.35%)		
High fever	1 (1.07%)	1 (1.03%)	6 (6.52%)		
Use of painkillers within 24 h after surgery [n(%)]				6.789	0.033
Using painkillers	18 (19.35%)^a^	33 (34.02%)^b^	32 (34.78%)^b^		
Not using painkillers	75 (80.65%)^a^	64 (65.98%)^b^	60 (65.22%)^b^		
Postoperative 0–24 h score $\left(\bar{x}\pm s\right)$	2.32 ± 1.29^a^	3.07 ± 1.47^b^	3.72 ± 1.49^c^	22.405	*P* < 0.001
WBC $\left(\bar{x}\pm s,\;10^{9}/L\right)$	8.33 ± 1.12	8.31 ± 1.56	8.58 ± 1.27	1.249	0.288
NEUT $\left(\bar{x}\pm s,\;10^{9}/L\right)$	4.75 ± 1.49	5.08 ± 1.39	4.67 ± 1.46	2.135	0.120
Hospital stay [M(P25,P75), d]	2.00 (1.00, 2.00)	2.00 (1.50, 2.00)	2.00 (1.00, 2.00)	4.013	0.134
Inpatient Costs $\left(\bar{x}\pm s,\;\mathrm{yuan}\right)$	13076.37 ± 3112.83	13820.46 ± 2678.02	13456.86 ± 2816.76	1.593	0.205

Please note the superscript letters (a, b, c) for each number; if the same letter appears, it indicates no statistical significance between them.

Following the implementation of the PSM algorithm, the short-term clinical safety profiles and recovery dynamics for balanced cohorts were comprehensively analyzed and are displayed in Table 6. Post-matching analyses confirmed that an elevated BMI remained tightly linked to a prolonged time to first flatus (*P* = 0.000), with recovery delayed stepwise from the Normal Weight Group (16.86 ± 2.31 h) to the Overweight Group (19.04 ± 2.63 h) and the Obesity Group (19.75 ± 2.58 h). For gastrointestinal complications, highly significant differences persisted regarding nausea and vomiting (*P* = 0.008) and abdominal distension (*P* = 0.001), with the Overweight Group (26.09% for nausea; 17.39% for distension) and the Obesity Group (28.57% for nausea; 22.54% for distension) tracking significantly higher than normal-weight pairs. Diarrhea events also remained significantly different (*P* = 0.006), concentrating primarily in the Obesity Group (16.33%). Coughing and expectoration remained statistically homogenous cross-groups (*P* = 0.171). Postoperative pyrexia patterns maintained statistical significance across the matched cohorts (*P* = 0.004), driven by a higher density of high-grade fever cases in the Obesity Group (10.20%). The requirement for rescue analgesic intervention within 24 h remained significantly greater in the elevated BMI pool (43.48% for overweight and 44.90% for obese) than in normal-weight counterparts (22.22%) (*P* = 0.018). The postoperative 0–24 h pain score was significantly lower in the Normal Weight Group (2.34 ± 1.30) than in the Overweight Group (3.35 ± 1.50) and the Obesity Group (3.63 ± 1.56) (*P* = 0.000). *Post-hoc* pairwise evaluations indicated that pain scores plateaued between the overweight and obese cohorts after balancing. Finally, postoperative day 1 laboratory parameters—comprising white blood cell count (*P* = 0.075) and neutrophil count (*P* = 0.350)—postoperative hospital stay (*P* = 0.438), and cumulative inpatient costs (*P* = 0.804) showed no statistical variations among the balanced sub-groups as documented in Table 6.

**Table 6:** Comparison of postoperative recovery and complications across body mass Index categories after propensity score matching.

Postoperative outcomes	Normal Weight Group (*n* = 72)	Overweight group (*n* = 23)	Obesity Group (*n* = 49)	Statistical test values	*P* value
First exhaust time after surgery $\left(\bar{x}\pm s,\;h\right)$	16.86 ± 2.31^a^	19.04 ± 2.63^b^	19.75 ± 2.58^b^	21.810	*P* < 0.001
Postoperative adverse reactions [n(%)]					
Nausea and vomiting	6 (8.33%)^a^	6 (26.09%)^b^	14 (28.57%)^b^	9.657	0.008
Coughing and expectoration	1 (1.39%)	1 (4.35%)	3 (6.12%)	3.437	0.171
Abdominal distension	2 (2.78%)^a^	4 (17.39%)^b^	11 (22.54%)^b^	12.452	0.001
Diarrhea	1 (1.39%)^a^	2 (8.70%)^a^	8 (16.33%)^b^	9.296	0.006
Postoperative fever occurrence [n(%)]				17.155	0.004
No fever	59 (81.94%)	16 (69.57%)	30 (61.22%)		
Low-grade fever	7 (9.72%)	4 (17.39%)	13 (26.53%)		
Moderate fever	5 (6.94%)	2 (8.70%)	1 (2.04%)		
High fever	1 (1.39%)	1 (4.35%)	5 (10.20%)		
Use of painkillers within 24 h after surgery[n(%)]				8.014	0.018
Using painkillers	16 (22.22%)	10 (43.48%)	22 (44.90%)		
Not using painkillers	56 (77.78%)	13 (56.52%)	27 (55.10%)		
Postoperative 0–24 h score $\left(\bar{x}\pm s\right)$	2.34 ± 1.30^a^	3.35 ± 1.50^b^	3.63 ± 1.56^b^	13.242	*P* < 0.001
WBC $\left(\bar{x}\pm s,\;10^{9}/L\right)$	8.24 ± 1.08	8.80 ± 1.42	8.66 ± 1.34	2.636	0.075
NEUT $\left(\bar{x}\pm s,\;10^{9}/L\right)$	4.97 ± 1.19	5.17 ± 1.07	5.29 ± 1.33	1.056	0.350
Hospital stay [M(P25,P75),d]	2.00 (1.00, 2.00)	2.00 (2.00, 2.00)	2.00 (1.00, 2.00)	1.651	0.438
Inpatient Costs $\left(\bar{x}\pm s,\;\mathrm{yuan}\right)$	13049.93 ± 3311.94	13477.72 ± 2738.58	13294.21 ± 2459.14	0.219	0.804

Please note the superscript letters (a, b) for each number; if the same letter appears, it indicates no statistical significance between them.

No wound infections, intra-abdominal abscesses, incisional hernias, or readmissions were reported during the one-month telephone follow-up among the followed cohort.

## 4 Discussion

This single-center retrospective study utilized a rigorous 1:1 propensity score-matched algorithm to elucidate the independent impact of stratified BMI on the perioperative profiles and recovery kinetics of patients undergoing SILA. The primary clinical findings demonstrate that while SILA remains a technically safe and highly feasible approach across all weight categories—evidenced by a zero conversion rate and homogenous post-matching laboratory inflammatory markers—an elevated BMI was associated with less favorable short-term operative and recovery profiles. Specifically, higher BMI was associated with a stepwise prolongation of operative duration, increased intraoperative blood loss, delayed return of gastrointestinal motility, and higher early postoperative pain scores. Crucially, however, these clinical differences were not accompanied by prolonged postoperative hospital stay or increased economic expenditures, supporting the safety and utility of SILA even in the obese segment.

### 4.1 Association of elevated BMI with intraoperative efficiency and technical challenges

The intraoperative data in this cohort reveal that an elevated BMI significantly prolongs operative duration (*P* = 0.002) and increases estimated surgical blood loss (*P* = 0.000), even after neutralizing anatomical confounders such as appendiceal length and width via PSM. In conventional multi-port laparoscopy, the utilization of standard triangulation helps overcome structural barriers in obese patients; however, the technical constraints of SILA are heavily magnified by abdominal adiposity.

Mechanistically, a thick abdominal wall dramatically increases the focal depth of the transumbilical multi-channel device, causing severe instrument crowding and restricted range of motion, commonly referred to as the “chopstick effect.” Furthermore, hyperplastic intra-abdominal and omental fat heavily compromises the structural visualization of the ileocecal region, demanding prolonged and repetitive retraction to achieve adequate skeletonization of the appendiceal base (20). The increased mechanical friction between laparoscopic tools within a single pivot point, coupled with the need for persistent monopolar electrocautery and clipping through abundant adipose tissue, accounts for the stepwise increase in operative time from 57.40 ± 16.82 min in normal-weight controls to 68.59 ± 17.33 min in obese individuals.

The statistically higher intraoperative blood loss observed in the Obesity Group (5.00 [5.00, 10.00] mL) further supports this anatomical challenge. Due to fat deposition flanking the mesoappendix, structural windows-opening and clean isolation of the appendiceal artery are significantly obscured. This anatomical ambiguity increases the risk of minor vascular tearing during blunt dissection, necessitating frequent electrocoagulation (21). Nevertheless, the absolute volume of blood loss remained within a highly acceptable clinical margin (<10 mL median), and the achievement of a zero middle-course conversion rate underscores that while obesity escalates the physical and technical workload of the operator, it does not compromise the fundamental safety barrier of the single-port laparoscopic approach.

### 4.2 Postoperative gastrointestinal recovery and adverse reactions

Post-matching outcomes demonstrated that an elevated BMI significantly impairs postoperative gastrointestinal motility restoration, characterized by a stepwise delay in the time to first flatus from 16.86 ± 2.31 h in the Normal Weight Group to 19.04 ± 2.63 h and 19.75 ± 2.58 h in the overweight and obese cohorts, respectively (*P* = 0.000). This slower functional return was observed alongside significantly higher rates of gastrointestinal complications, including nausea and vomiting (*P* = 0.008) and abdominal distension (*P* = 0.001).

Physiologically, the prolonged insufflation of carbon dioxide pneumoperitoneum and extensive mesenteric manipulation required for obese patients may trigger a local sympathetic neurogenic reflex, which could inhibit intestinal smooth muscle contractility (22, 23). Furthermore, hypertrophic intra-abdominal adipose tissue undergoes enhanced localized lipolysis under surgical stress, releasing excess free fatty acids and pro-inflammatory cytokines (such as TNF-*γ* and IL-6) into the peritoneal microenvironment (24). This micro-inflammatory cascade may exacerbate localized intestinal wall edema, potentially delaying electrical-mechanical coupling within the enteric nervous system (25).

### 4.3 Acute postoperative pain intensity and analgesic consumption

Quantified data illustrated that acute post-surgical pain profiles were highly exacerbated by increased body weight, with the 0–24 h pain score rising from 2.34 ± 1.30 in normal-weight controls to 3.35 ± 1.50 in overweight and 3.63 ± 1.56 in obese patient categories (*P* = 0.000). Consequently, the objective requirement for rescue painkillers within the first 24 h expanded to 43.48% and 44.90% in the elevated BMI segments (*P* = 0.018).

This elevated pain perception may be primarily related to the biomechanical demands of single-port platforms. Due to the marked thickness of the subcutaneous fat layer flanking the umbilicus, the single pivot port experiences increased resistance during tool manipulation. To achieve satisfactory exposure of the retrocecal or deeply situated appendix, the operator must exert greater physical leverage and torque on the device, causing intensive mechanical stretching, friction, and focal ischemia of the umbilical fascial rings and dermal margins (26). This continuous somatic traction triggers dense nociceptive signaling via peripheral mechanoreceptors (27).

Additionally, the substantial volume of adipose tissue divided by monopolar electrocautery may lead to wider thermal dissipation zones and lipid necrosis, which could release a high concentration of prostaglandins and bradykinin into the incision site, potentially lowering the activation threshold of localized pain pathways (28, 29). Despite these challenges, the implementation of a standardized on-demand analgesic strategy—relying on the selective single-dose administration of either a Class II psychotropic agent (tramadol hydrochloride) or a targeted injectable NSAID (ketorolac tromethamine) tailored to individual pain severity—successfully mitigated breakthrough pain events, preventing pain scores from escalating into the severe tier (> 5 points).

### 4.4 Systematic inflammation, postoperative hospital stay, and economic efficiency

An insightful finding of this study is that despite the increases in operative duration, pain intensity, and transient gastrointestinal delays, the acute inflammatory responses and economic efficiency indices were comparable across all cohorts. Postoperative day 1 white blood cell counts (*P* = 0.075) and neutrophil fractions (*P* = 0.350) achieved total homogeneity, indicating that the surgical trauma scale of SILA does not amplify systemic immune-inflammatory cascades in obese patients. Furthermore, although the Obesity Group demonstrated a higher density of high-grade fever occurrences (10.20%, *P* = 0.004), this pyrexial reaction may reflect a sterile metabolic stress response related to adipose thermal lipolysis rather than active infectious sequelae, as suggested by the non-elevated inflammatory lab panels.

Crucially, the postoperative hospital stay (*P* = 0.438) and cumulative inpatient costs (*P* = 0.804) were statistically identical among the matched sub-groups. This macroeconomic equilibrium highlights the profound clinical value of integrating an evidence-based ERAS pathway. By implementing early ambulation, micro-tailored fluid preservation, and non-opioid multimodal analgesia, the early physiological delays associated with high BMI were successfully contained within the immediate 24 h phase (30, 31). Consequently, the structural advantages of SILA—such as reduced wound complications and rapid systemic rehabilitation—were fully preserved, suggesting that increased body mass was not associated with compromised overall efficiency of health resource utilization or prolong hospital convalescence.

### 4.5 Limitations

Several limitations should be acknowledged. First, the retrospective, single-center design may introduce selection bias and limits the generalizability of the findings to other settings, although bias was mitigated by propensity score matching. Second, the small overweight subgroup (*n* = 23) limits statistical power; accordingly, the primary comparison remains between the normal-weight and obese groups. Third, clinical severity indicators such as perforation, abscess, or peritonitis were not systematically recorded, and residual confounding from unmeasured severity cannot be excluded. Fourth, long-term outcomes including scar cosmesis and incisional hernia were not evaluated. Fifth, as demonstrated in laparoscopic cholecystectomy cohorts, the effect of BMI on postoperative outcomes may vary according to age and overall risk profile (32); however, the present sample size precluded meaningful age-stratified or interaction analysis. Future prospective studies with standardized severity grading, extended follow-up, and adequate power for age-BMI interaction analysis are warranted.

## 5 Conclusions

In conclusion, an elevated BMI was associated with greater technical difficulty in SILA, including prolonged operative duration, increased intraoperative blood loss, delayed return of gastrointestinal motility, and higher acute postoperative pain scores. Although these physiological differences may not compromise core safety outcomes, prolong postoperative hospital stays, or elevate inpatient costs, the findings suggest that caution is warranted when selecting obese patients for SILA, and that individualized perioperative management should be considered. While obesity is not an absolute contraindication for SILA, careful patient selection and optimized perioperative pathways are advisable to mitigate acute postoperative gastrointestinal distress and somatic pain in this population.

## Data Availability

The datasets utilized and/or analyzed during the current study are available from the corresponding author upon reasonable request. Requests to access the datasets should be directed to 17603113060@163.com.
